# Molecular Characterization and Cluster Analysis of Field Isolates of Avian Infectious Laryngotracheitis Virus from Argentina

**DOI:** 10.3389/fvets.2017.00212

**Published:** 2017-12-13

**Authors:** María I. Craig, Maria F. Rojas, Claudia A. van der Ploeg, Valeria Olivera, Ariel E. Vagnozzi, Andrés M. Perez, Guido A. König

**Affiliations:** ^1^Instituto de Virología, Centro de Investigación en Ciencias Veterinarias y Agronómicas, Instituto Nacional de Tecnología Agropecuaria (INTA)-Hurlingham, Buenos Aires, Argentina; ^2^Laboratorio de Sanidad Aviar, Estación Experimental Agropecuaria, Instituto Nacional de Tecnología Agropecuaria (INTA)-Concepción del Uruguay, Entre Rios, Argentina; ^3^Instituto Nacional de Producción de Biológicos, ANLIS “Dr. Carlos G. Malbrán”, Ciudad de Buenos Aires, Argentina; ^4^Department of Veterinary Population Medicine, College of Veterinary Medicine, University of Minnesota, Saint Paul, MN, United States; ^5^Instituto de Biotecnología, Centro de Investigación en Ciencias Veterinarias y Agronómicas INTA-Hurlingham, Buenos Aires, Argentina; ^6^Consejo Nacional de Investigaciones Científicas y Técnicas (CONICET), Buenos Aires, Argentina

**Keywords:** infectious laryngotracheitis virus, molecular characterization, epidemiology, spatial cluster analysis, glycoprotein J, Argentina

## Abstract

Avian infectious laryngotracheitis (ILT) is a worldwide infectious disease that causes important economic losses in the poultry industry. Although it is known that ILT virus (ILTV) is present in Argentina, there is no information about the circulating strains. With the aim to characterize them, seven different genomic regions (thymidine kinase, glycoproteins D, G, B, C, and J, and infected cell polypeptide 4) were partially sequenced and compared between field samples. The gJ sequence resulted to be the most informative segment, it allowed the differentiation among field sample strains, and also, between wild and vaccine viruses. Specific changes in selected nucleotidic positions led to the definition of five distinct haplotypes. Tests for detection of clustering were run to test the null hypothesis that ILTV haplotypes were randomly distributed in time in Argentina and in space in the most densely populated poultry region of this country, Entre Rios. From this study, it was possible to identify a 46 km radius cluster in which higher proportions of haplotypes 4 and 5 were observed, next to a provincial route in Entre Rios and a significant decline of haplotype 5 between 2009 and 2011. Results here provide an update on the molecular epidemiology of ILT in Argentina, including data on specific genome segments that may be used for rapid characterization of the virus in the field. Ultimately, results will contribute to the surveillance of ILT in the country.

## Introduction

Infectious laryngotracheitis (ILT) is an acute and highly contagious respiratory disease of chicken that causes far-reaching financial losses due to its high mortality rate and reduction in egg production. Chickens can be infected through the upper respiratory and ocular routes (1). The ILT etiological agent, generically referred to as ILT virus (ILTV) and also known as *Gallid herpesvirus 1*, is a member of the *Herpesviridae* family with a double-stranded DNA of approximately 155 kbp in size (2).

Traditionally, two types of ILTV attenuated vaccines have been widely used to control the disease, namely, chicken embryo-origin (CEO) vaccine, which is attenuated by serial passages in embryonated eggs (3), and a tissue culture-origin (TCO) vaccine, which is generated by multiple passages in tissue culture (4). Since 2006, use of the CEO vaccine has been banned in Argentina, and currently, TCO and two vector vaccines, which express the gB gene linked, are commercially available in the country.

The ILTV is globally distributed and several epidemiological studies have been conducted in different countries for detecting circulating ILTVs. Traditionally, differentiation of wild from vaccine strains has been based on the use of restriction length polymorphism (RFLP) profiles of the complete genome (5–8). Recently, however, amplification by polymerase chain reaction (PCR) combined with RFLP of different regions of the ILTV genome [timidine kinase, glycoprotein G, E, X, and C (gG, gE, gX, and gC) and infected cell polypeptide 4 (ICP4)] has been used to characterize strains, with varying degrees of success (9–12). Even in the absence of multiple ILTV serotypes, molecular characterizations is still epidemiologically useful to assess the virus dissemination process in a region.

Infectious laryngotracheitis virus occurs in Argentina sporadically and to our best knowledge, no evaluation of its prevalence and no characterization of field viruses have been conducted. Thus, considering the absence of information on ILTV strains circulating, the purpose of the study here was to assess the molecular epidemiology of ILTV, using sequences from the most variable regions of the genome corresponding to proteins with antigenic or functional relevance (TK, gD, gG, gB, gC, gJ, and ICP4). Results will help select the most variables ILTV genome regions, characterize field strains, and differentiate ILTV field and vaccine strains.

## Materials and Methods

### Clinical Samples

Field strains (*n* = 72) were obtained from the upper respiratory tract (tracheas or tracheal swabs) of broilers and layers showing mild to severe disease signs during different outbreaks between 2006 and 2013. The samples belong to 12 localities in Buenos Aires (there are nine samples with unknown location) and from 15 in Entre Rios province. Samples were spontaneously submitted to the diagnostic laboratory for disease testing and confirmation by producers that suspected the presence of the disease to one of two different INTA (Instituto Nacional de Tecnología Agropecuaria) avian virology laboratories, located in Buenos Aires and Entre Rios provinces.

### Sample Collection and DNA Extraction

The DNA was extracted either from samples of tracheal mucosa or directly from tracheal swabs. Tracheal mucosa were scraped and homogenized in a mortar with sterile sand and PBS supplemented with penicillin 10,000 IU/ml, streptomycin 5,000 µg/ml, gentamicin sulfate 1,000 µg/ml, kanamycin sulfate 700 µg/ml, and amphotericin B 10 µg/ml (Sigma Chemical Co™, St. Louis, MO, USA); subsequently, centrifugation was performed to eliminate cell debris and sand. The supernatant was collected and stored at −70°C.

Tracheal swabs were obtained by a strong rubbing of the mucosal zone and were suspended in 2 ml of sterile PBS. DNA was extracted from the supernatants and reconstituted vaccines Laringo-Vac (CEO—Solvay Animal Health) and LT-IVAX (TCO—Schering Plough.) by QIAamp DNA Mini Kit (Qiagen Inc., Valencia, CA, USA), according to the manufacturer’s instructions.

### Polymerase Chain Reaction

Primers for the gB and the gD were designed with Fast_PCR software version 3.3.64 (13), on the basis of sequences previously published in Genbank. The rest of the primers were obtained from published data (Table 1).

**Table 1:** Set of primers and cycling conditions for the amplification of different genomic regions of ILT virus.

Name	Target genes	Sequence (5′–3′)	Polymerase chain reaction (PCR) cycle conditions	Approximate size (kbp) of expected PCR product	Reference
gB-For	gB	TTCACTATAGGCTGGGATGCA	94 C (2 min), 35 × (94 C 30 seg; 55 C 30 seg; 72 C 1.5 min) 72 C 5 min	1.55	–^a^
gB-R		TGGCAAGTATCCTGTCGTCCT			

gD-For	gD	AGCAGGCGAGGCGTGGATTTC	94 C (2 min), 35 × (94 C 30 seg; 62 C 30 seg; 72 C 1 min) 72 C 5 min	1.1	–^a^
gD-R		CCGAGTCTTCTGGAGGGGCCT			

gG-For	gG	CCTTCTCGTGCCGATTCAATATG	94 C (2 min), 35 × (94 C 30 seg; 55 C 30 seg; 72 C 1.5 min) 72 C 5 min	1.48	Kirkpatrick et al. (10)
gG-R		AACCACACCTGATGCTTTTGTAC			

gJ-For	gJ	ATTTCGCCGAGAGATGGGGAC	94 C (2 min), 35 × (94 C 30 seg; 53 C 30 seg; 72 C 1.5 min) 72 C 5 min	1.39	Veits et al. (14)
gJ-R		CAGTGTATTTTCTGACTCACCG			

gC-For	gC	AACATGCAGCATCAGAGTACTG	94 C (2 min), 35 × (94 C 30 seg; 54 C 30 seg; 72 C 1.5 min) 72 C 5 min	1.26	Veits et al. (14)
gC-R		CGTTTATGTTGTCTTCCAGCAC			

TK-For	TK	CTGGGCTAAATCATCCAAGACATCA	94 C (2 min), 35 × (94 C 30 seg; 55 C 30 seg; 72 C 1.5 min) 72 C 5 min	2.24	Kirkpatrick et al. (10)
TK-R		GCTCTCTCGAGTAAGAATGAGTACA			

ICP4-2F	ICP4	CTTCAGACTCCAGCTCATCTG	94 C (3 min), 35 × (94 C 1 min; 62 C 1 min; 72 C 1.5 min) 72 C 10 min	0.63	Chacón et al. (12)
ICP4-2R		AGTCATGCGTCTATGGCGTTGAC			

*^a^Sets of primers designed by Fast_PCR software version 3.3.64*.

DNA amplification was carried out in 50 µl reaction volume using 4 µl of extracted DNA, 5 µl of 10× reaction buffer, 1 µl of 10 mM dNTPs mix, 1 µl of each primer (5 µmol), and 0.25 µl of 5 U/μl of GoTaq DNA Polymerase (Promega). The cycling program for each amplification is described in Table 1.

### Amplicon Purification and Sequence Analysis

The amplicons were purified by QIAquick PCR purification kit (Qiagen Inc., Valencia, CA, USA), according to the manufacturer’s instructions.

Sequences of each amplicon were directly sequenced with the Big Dye terminator kit TM (Applied Biosystems, Foster City, CA, USA) on an ABI 3130 or 3500 XL TM (Applied Biosystems, Foster City, CA, USA).

Sequences were edited using the BIOEDIT software (15). Sequences obtained here and reference strains sequences obtained from Genbank were included in a multiple alignment with CLUSTAL W program Version 1.8.3 (16).

### Space and Time Cluster Analysis

The observed to expected case ratio (o/e), where the expected number of cases was computed under the null hypothesis of random distribution of cases, was estimated. A spatial multinomial scan statistic was implemented to assess whether there were areas in which the proportion of some haplotypes was significantly different to the rest of the study area. A temporal multinomial scan statistic was also carried out to assess whether there were periods when the proportion of some haplotypes was significantly different to the rest of the study period. Both analysis were performed in the SaTScan V.8.0 software (17). Clustering was assessed using a multivariate model and procedures described elsewhere (18, 19).

## Results

### Sample Distribution

Most (93%) of the 72 field samples were collected from the two provinces in which most of the poultry production is concentrated in Argentina, namely, Entre Rios (*n* = 39 samples, 54.2%) and Buenos Aires (*n* = 28 samples, 38.8%). Samples became from 20 flocks distributed in 12 localities in Buenos Aires and 32 flocks from 15 localities in Entre Rios province. Unfortunately, geolocalization from all Buenos Aires and 13 Entre Rios samples were not provided. The remaining 7% (*n* = 5) of the samples were collected in others provinces like Cordoba, Neuquén, Rio Negro, and Mendoza.

### Selection of the Target Genomic Region

With the objective of selecting the most appropriate genomic sequence/s for epidemiological studies on circulating ILTV strains, many PCRs were performed on different genomic regions, including TK, ICP4, gC, gD, gB, gG, and gJ. Initially, the analysis was conducted on both types of attenuated vaccines (CEO and TCO) and 17 field samples.

Analysis of the amplicon sequences corresponding to TK and gC genes did not show differences among field samples or even between the vaccines.

Sequence alignment were compared to complete sequences available in the Genebank. Although the analysis of the ICP4 sequence allowed to clearly distinguishing between TCO and CEO vaccines, there were no differences between the CEO vaccine and field isolates. In contrast, gB showed two changes at positions 1043 (T/C) and 1931 (C/T), and those changes allowed distinguishing between TCO and CEO vaccines, and between vaccines and fields samples, although no differences were found among the latter (Table 2).

**Table 2:** Positions of nucleotide changes in amplicons of different genomic regions and defined haplotype according to gJ amplified sequence.

Nucleotide position from ATG	Infected cell polypeptide 4^a^						gB^b^		gD^c^	gG^d^	gJ^e^					Haplotype
			
3875	3927	3951	3982	4017	4309	1043	1931	163	316	461	484	832	878	894
ER07_01	T	C	C	A	A	T	T	C	T	T	A	C	G	T	G	5
ER08_03	T	C	C	A	A	T	T	C	C	T	A	C	A	T	A	4
ER08_05	T	C	C	A	A	T	T	C	C	T	A	C	A	T	A	4
ER08_07	T	C	C	A	A	T	T	C	C	T	A	C	G	T	G	5
ER08_06	T	C	C	A	A	T	T	C	C	T	A	C	G	T	G	5
ER09_02	T	C	C	A	A	T	T	C	C	T	A	C	A	T	A	4
ER09_03	T	C	C	A	A	T	T	C	C	T	A	C	A	T	A	4
RN09_09	T	C	C	A	A	T	T	C	C	T	A	C	A	T	G	2
ER10_01	T	C	C	A	A	T	T	C	C	T	T	C	A	T	G	3
ER08_01	T	C	C	A	A	T	T	C	C	T	A	C	G	T	G	5
ER06_01	T	C	C	A	A	T	T	C	C	T	A	C	A	T	G	2
BA09_07	T	C	C	A	A	T	T	C	C	T	T	C	A	T	G	3
RN10_06	T	C	C	A	A	T	T	C	C	T	A	C	A	T	G	2
ER06_02	T	C	C	A	A	T	T	C	C	T	A	C	A	T	G	2
ER06_03	T	C	C	A	A	T	T	C	C	T	A	C	G	T	G	5
TCO^f^	C	T	T	G	G	A	C	T	C	G	A	T	A	C	G	1
CEO^f^	T	C	C	A	A	T	T	T	C	G	A	C	A	T	G	2

*The position of nucleotides changes was defined by the comparison to complete codons sequences available in the Genebank*.

*The identification code for samples (LLNNXXX) is: first two letters (LL) for the province, followed by two numbers for the year (NN), and the rest for sample identification*.

*^a^EU104900.1*.

*^b^EU104973.1*.

*^c^JN580317.1*.

*^d^JN969106.1*.

*^e^JN969108.1*.

*^f^Vaccines: CEO, Chicken embryo origin; TCO, Tissue Culture Origin*.

*BA, Buenos Aires; ER, Entre Rios; RN, Rio Negro*.

Different shades indicate different haplotypes

In gD and gG amplicons, only one position changed, 163 (C/T) and 316 (T/G), respectively. The nucleotide change detected in gD sequence was observed in only one field sample and the others showed no changes between them and the vaccines. The gG sequence was able to differentiate vaccines from field samples, but could not differentiate among field samples or between vaccines (Table 2).

Analysis of the amplified gJ region evidenced five changes, in positions 461 (A/T), 484 (C/T), 832 (G/A), 878 (T/C), and 894 (G/A), which permitted to differentiate between vaccines and among field strains. The combination of these nucleotide differences determined 5 haplotypes, numbered from 1 to 5 (Table 2).

Only gJ sequence was used to characterize the rest (*n* = 55) of the samples, in addition to the 17 samples initially assessed.

### Glycoprotein J Sequence Analysis

Haplotypes 1, 2, and 3 were found in Buenos Aires, and haplotype 2 was also detected in other regions (Table 3).

**Table 3:** Presence of haplotypes in Buenos Aires and other provinces.

Sample	Haplotype	Accession *n*
**TCO^a^**	1	JN580312.1
BA10_149_647	1	MF443785
BA10_149_672	1	MF443786
**CEO^a^**	2	KP677881.1
BA10_141	2	MF443787
BA10_154(740)	2	MF443788
BA10_154 (741)	2	MF443789
BA10_152	2	MF443790
BA09_068	2	MF443791
BA11_195 (300)	2	MF443792
BA11_195 (349)	2	MF443793
BA11_176	2	MF443794
BA12_210	2	MF443795
BA12_224 (GNA)	2	MF443796
NQ09_103	2	MF443797
RN09_109 (9)	2	MF443798
RN10_33(A)	2	MF443799
Mza 12_203	2	MF443800
Cba 12_245	2	MF443801
BA 11_156	3	MF443802
BA10_111	3	MF443803
BA09_ 096	3	MF443804
BA10_149 (675)	3	MF443805
BA09_092	3	MF443806
BA09_071	3	MF443807
BA08_057	3	MF443808
BA11_195 (304)	3	MF443809
BA11_180	3	MF443810
BA08_051	3	MF443811
BA09_099.397	3	MF443812
BA09_097	3	MF443813
BA09_099.403	3	MF443814
BA11_163	3	MF443815
BA11_196	3	MF 443816
BA12_223(GHIC)	3	MF443817

*^a^Vaccines: CEO, Chicken embryo origin; TCO, Tissue Culture Origin*.

*BA, Buenos Aires; NQ, Neuquen; RN, Rio Negro; Mza, Mendoza; Cba, Cordoba*.

**Figure 1:**
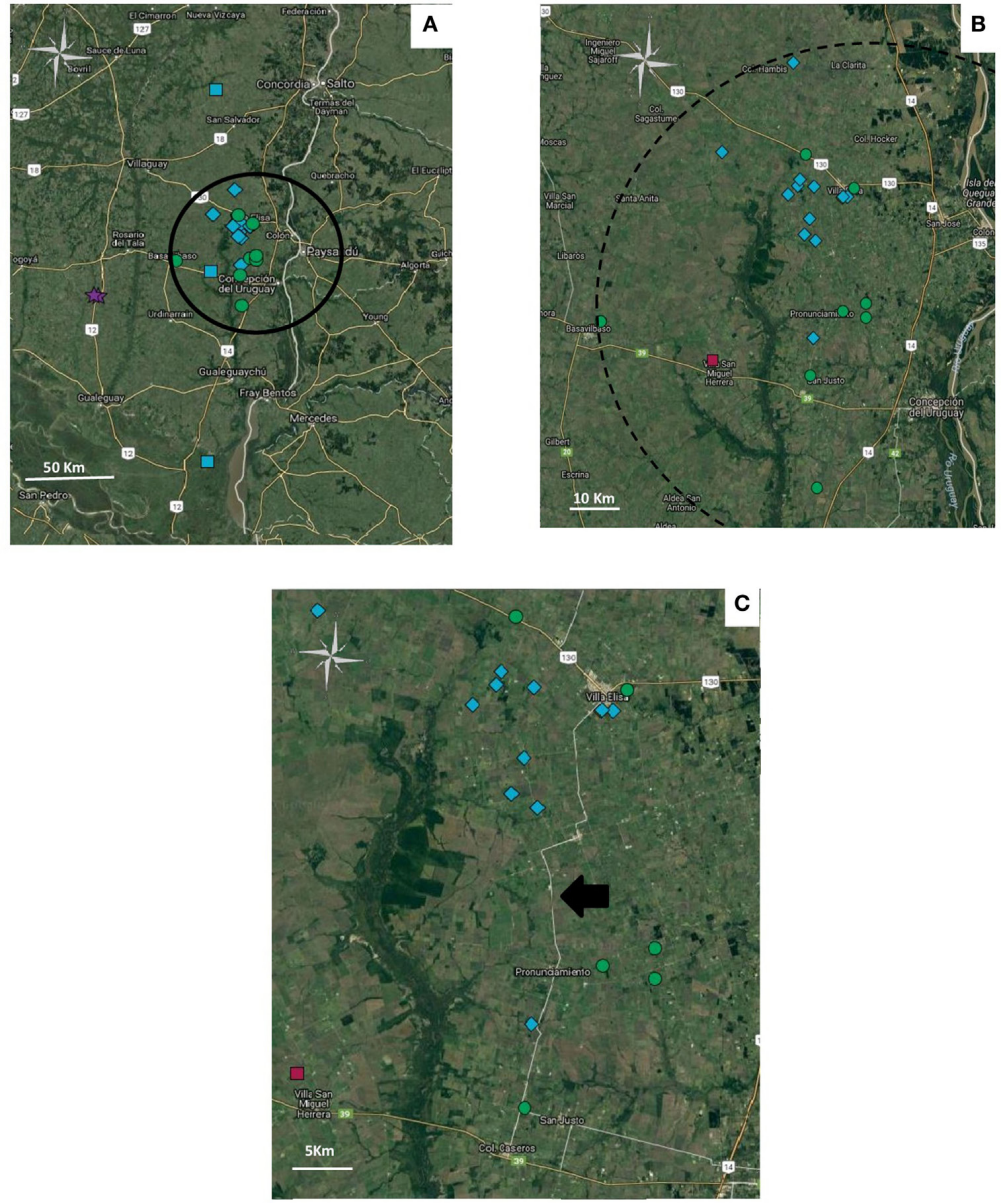
Geolocation of the haplotypes in Entre Rios province. Haplotypes: 2 (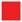), 3 (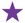), 4 (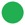) y 5 (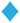). **(A)** Distribution of geolocated haplotypes in Entre Rios Province. Cluster is indicated as a circle. **(B)** Amplification of the zone where haplotype 5 and 4 are concentrated. **(C)** Distribution of haplotypes 4 and 5 along provincial route 23, pointed out with a black arrow. Figure was created with Google.

The five haplotypes were circulating in Entre Rios, the most densely populated poultry region and from where most (*n* = 39) samples were collected, and haplotypes 4 and 5 were found only in Entre Rios (Table 4). Interestingly, haplotypes 4 and 5 were mostly detected in different farms concentrated in an area of 6.600 km^2^ located in the Concepción del Uruguay and Colón counties, most of them were located near a road, referred to as provincial route 23 (Figure 1).

**Table 4:** Haplotypes and geolocation in Entre Rios province.

Sample	Haplotype	Geolocation		Accession n
ER11_162	1	nd	Nd	MF443818
ER08_11	2	nd	Nd	MF443819
ER06_02	2	−33,447748	−58,665275	MF443820
ER08_04	2	nd	Nd	MF443821
ER06_01	2	−32,413150	−58,645810	MF443822
		−31,414980	−58,612490	
ER09_01	3	−32,550469	−59,326217	MF443823
ER09_08	3	−32,544914	−59,353658	MF443824
ER13_14	3	nd	nd	MF443825
ER10_01	3	nd	nd	MF443826
ER09_86	4	nd	nd	MF443827
ER08_03	4	−32,350390	−58,362760	MF443828
ER08_05	4	−32,330250	−58,362500	MF443829
ER09_02	4	−32,602190	−58,452790	MF443830
ER09_03	4	−32,436272	−58,465756	MF443831
ER12_07	4	−32,109806	−58,472714	MF443832
ER12_14	4	−32,158847	−58,384447	MF443833
ER12_77	4	−32,341494	−58,404097	MF443834
ER12_81	4	−32,357272	−58,852136	MF443835
ER06_03	5	nd	nd	MF443836
ER07_01	5	nd	nd	MF443837
ER07_02	5	nd	nd	MF443838
ER08_01	5	nd	nd	MF443839
ER12_63	5	−32,155364	−58,488197	MF443840
ER12_66	5	−32,146375	−58,484261	MF443841
		−32,157286	−58,458553	
		−31,972681	−58,496206	
ER12_67	5	−32,172242	−58,404817	MF443842
ER12_75	5	nd	nd	MF443843
ER12_84	5	−32,168869	−58,506967	MF443844
ER12_85	5	−32,105856	−58,628931	MF443845
ER08_06	5	−32,228160	−58,476050	MF443846
ER08_07	5	−32,204339	−58,466733	MF443847
ER08_27	5	−32,237425	−58,455722	MF443848
ER08_30	5	−32,172428	−58,396044	MF443849
ER09_09	5	−32,380572	−58,460018	MF443850
ER10_25	5	−32,172844	−58,405578	MF443851
ER13_21	5	nd	nd	MF443852
ER13_22	5	nd	nd	MF443853

*ER, Entre Rios; nd, non determined*.

The analysis of the deduced amino acid composition of gJ sequence showed differences among haplotypes. Nucleotides changes in haplotype 4 were synonymous respect to haplotype 2 (showing an identical amino acid composition); however, haplotype 1 showed changes in two positions, (W/R) 163 and (A/V) 293, whereas haplotype 3 and 5 showed differences in position, (I/N) 154 and (G/S) 278, respectively.

### Cluster Analysis

The spatial multinomial scan was carried out only in the Entre Rios province, which concentrates most of the poultry production of the country and also most (*n* = 39) of samples collected, because there were too many missing locations from the rest of the study area. As 13/39 farm geolocations from the Entre Rios province were missing, the analysis was carried out on the remaining 26 samples collected. The temporal multinomial scan was carried out using the entire study area (72 samples).

In the analysis of spatial clustering centered in Entre Rios, only a cluster of 46 km radius was detected, in which a higher proportion of observed/expected cases of haplotype 4 and 5 cases (o/e = 2.22 and 2, 22, respectively) showed that the distribution of haplotypes was not randomized (Table 5).

**Table 5:** Spatial clustering in the Entre Rios region and temporal clustering over the entire study area and period.

Cluster	*n*	Temporal distribution	Spatial distribution	Observed/expected ratio					*P* value
				H1	H2	H3	H4	H5	
Entre Rios	26		Lat: 32.35039 S	0.74	0.15	0	2.22	2.22	<0.001
			Long: 58.362760 W						
			Radius: 45.95 km						

Argentina	72	2009–2011		2.06^a^	1.13	1.65	0.69	0.21	<0.001

*The name of the cluster indicates the region where it was detected. The observed/expected ratio of every haplotype is stated*.

*^a^The low number of observed/expected cases (3/1.46) could bias this result*.

Results of the time cluster analysis suggested a remarkable decline of haplotype 5 detection during the second half of the study period (2009–2011). Overall distribution of haplotypes was significantly different (*p* < 0,001) for those 3 years compared to that expected under the null hypoteshsis of even distribution of haplotypes (o/e ratio of 2.06, 1.13, 1.65, 0.69, 0.21 for haplotype 1–5, respectively) (Table 5).

## Discussion

Five haplotypes of circulating ILTV in Argentina were identified. Although the analysis by RFLP in different regions of the genome was successfully used by others to characterize ILTV strains (9–12), there is evidence that differentiation of field isolates from vaccine strains varies depending on the region of the world assessed (20). There are differences, not only in the regions of the genome analyzed but also in the number of these region used to characterized field strains and vaccines (20). Certainly, the analysis of several regions would be better to characterize the circulating strains as it would provide more informative sites and also have the potential to detect the presence of recombinants. Recently, a multi-allelic PCR-sequencing analysis proved to be an efficient tool in differentiating ILTV strains globally (21). However, we found that two of the assayed regions (ICP4 and gB) did not add more discriminative information than gJ alone, at least for Argentine strains. So, among the seven regions under study here, gJ resulted the most variable on region, determining 5 variants or haplotypes circulating in the country.

Haplotype 1 covers the TCO vaccine and few Argentine field strains. The comparison of these sequences and several sequences from the Genbank showed that nucleotide T in position 484 and nucleotide in position C 878 are present both in the TCO vaccine and strain 81658 (JN542535), which may have been acquired from chickens vaccinated with TCO (22), and also in the USDA reference strain (JN542534), which showed very similar RFLP pattern when compared to the TCO (23). Presence of haplotype 1 in the field strains reported here may have been related to the finding by chance, of the TCO strain in recently vaccinated animals with a compromised immune state. Although we have no vaccine information related to the isolates of Buenos Aires (BA149-647 and 672), it is likely that animals from Entre Rios (ER 11-162) may have been vaccinated with the TCO vaccine.

Haplotype 2, which included the CEO vaccine, seems to be widely spread through the country. The origin of this haplotype in Argentina could probably be related with the introduction of the CEO vaccine. The use of the CEO vaccine has been banned in Argentina for the last 10 years, because of its associated reversion to virulence (1). However, it is likely that the CEO vaccine strain may have still been circulating since then, reverting to its virulent form to produce new outbreaks. However, such hypothesis requires further analysis, based on sequences of other ILTV genome regions, to test the homology of those field samples with the CEO strain.

Comparison of other ILTV field sequences here to other gJ sequences obtained from the Genebank suggests that the presence of certain nucleotides at positions 461 (**T**), 832 (**G**), and 894 (**A**), are exclusive to the Argentinian strains. Those changes are responsible for defining the circulating haplotypes 3, 5, and 4, respectively.

Although the analysis of the gJ sequence was not comprehensive enough to completely characterize circulating strains, differentiation of at least some of the circulating field strains was possible. Such characterization helped to identify and differentiate widespread from locally circulating strains. Haplotypes 4 and 5 were more prevalent in Entre Rios, compared to Buenos Aires. Moreover, most haplotype 4 and 5 cases were concentrated along a 46 km radius, on an area with high density of poultry farms located nearby a route (provincial route 23) frequently used by trucks used to move poultry. Data also suggest that both haplotypes have been circulating in Entre Rios since, at least, 2006 and 2008, respectively. That observation, in addition to the small, restricted area where those haplotypes coexist, suggests that some factors, like suppliers or shared workers, could be implicated in the transmission, maintenance, and high prevalence of these haplotypes in such limited region.

A remarkable drop of the frequency of haplotype 5 was detected during 2009–2011, followed by its recovery in 2012. Although, it is difficult to rule out that the reduction in incidence of haplotype 5 may have been due to selective under-reporting or under-detection during the study period, no data supports these explanations.

Alternative explanations may also include the reintroduction of the haplotype, after initial extinction, from an external source, or a variation on the virus virulence and transmissibility.

The analysis of deduced amino acid composition presented changes that affected not only the structure but also polarity of the virus, in some haplotypes. The possible effect of those changes on the gJ structure, also on its function, is unknown. Further functionality or structural studies on gJ would be necessary to analyze the relevance of those changes.

In the event of absence of complete or several partial sequences of ILTV strains, the methodology here may help to support the initial classification of ILTVs, and routine monitoring of changes in the pattern of prevalent haplotypes, which may ultimately help to assess the epidemiological situation of the disease in Argentina, and other regions endemically affected by the ILTV.

Nevertheless, taking into account, the reported allelic variation presented in other genomic regions (21) than the one studied here, further analysis of these regions would allow us to further differentiate field samples and compare them to circulating strains in other countries.

## Author Contributions

GK and MC conceived and designed the study. CP, MR, and VO were responsible for most of PCR assay as well as data edition and the acquisition of field samples. GK, MC, AP, CP, FR, and VO contributed in data analysis. GK, MC, AP, and AV were responsible for data interpretation. The manuscript was written by MC, GK, AP, and also participated in the final revision of the manuscript as well as AV.

## Conflict of Interest Statement

The authors declare that the research was conducted in the absence of any commercial or financial relationships that could be construed as a potential conflict of interest.
